# The Potential of Menstrual Blood-Derived Mesenchymal Stem Cells for Cartilage Repair and Regeneration: Novel Aspects

**DOI:** 10.1155/2018/5748126

**Published:** 2018-12-03

**Authors:** I. Uzieliene, G. Urbonaite, Z. Tachtamisevaite, A. Mobasheri, E. Bernotiene

**Affiliations:** ^1^Department of Regenerative Medicine, State Research Institute Centre for Innovative Medicine, Vilnius LT-08406, Lithuania; ^2^Department of Veterinary Pre-Clinical Sciences, School of Veterinary Medicine, Faculty of Health and Medical Sciences, University of Surrey, Guildford GU2 7AL, UK; ^3^Arthritis Research UK Centre for Sport, Exercise and Osteoarthritis, Queen's Medical Centre, Nottingham NG7 2UH, UK

## Abstract

Menstrual blood is a unique body fluid that contains mesenchymal stem cells (MSCs). These cells have attracted a great deal of attention due to their exceptional advantages including easy access and frequently accessible sample source and no need for complex ethical and surgical interventions, as compared to other tissues. Menstrual blood-derived MSCs possess all the major stem cell properties and even have a greater proliferation and differentiation potential as compared to bone marrow-derived MSCs, making them a perspective tool in a further clinical practice. Although the potential of menstrual blood stem cells to differentiate into a large variety of tissue cells has been studied in many studies, their chondrogenic properties have not been extensively explored and investigated. Articular cartilage is susceptible to traumas and degenerative diseases, such as osteoarthritis, and has poor self-regeneration capacity and therefore requires more effective therapeutic technique. MSCs seem promising candidates for cartilage regeneration; however, no clinically effective stem cell-based repair method has yet emerged. This chapter focuses on studies in the field of menstrual blood-derived MSCs and their chondrogenic differentiation potential and suitability for application in cartilage regeneration. Although a very limited number of studies have been made in this field thus far, these cells might emerge as an efficient and easily accessible source of multipotent cells for cartilage engineering and cell-based chondroprotective therapy.

## 1. Introduction

Mesenchymal stem cells (MSCs) with their multipotent differentiation capability attract a lot of attention from researchers, developing possible ways of employing these cells in clinical practice. MSCs have been isolated and studied from different sources, including bone marrow, adipose tissue, synovial membrane, umbilical cord, and dental pulp [1]. The bone marrow is the primary tissue where MSCs were firstly isolated in 1957 and is considered to be a classical MSC source, which is often used as a control for other source MSCs [2].

In 2007, Meng with colleagues isolated a MSC population from menstrual blood (MenSC) [3]. MSC properties, including multiple differentiation, have been confirmed for these cells, while their differentiation capability and multipotency were even greater than bone marrow-derived MSCs (BMMSCs), suggesting that MenSCs are potent candidates for clinical applications. Furthermore, MenSCs are much easier to access compared to BMMSCs as their collection does not require complicated ethical procedures or any invasive surgical interventions, thus providing an option of repeated sample collection in the same donor. These advantages suggest MenSCs as an attractive tool for regenerative medicine.

Articular cartilage is an avascular load-bearing connective tissue with unique mechanical properties. However, the cartilage is a poor self-regenerating tissue and is highly susceptible to trauma or degenerative diseases such as osteoarthritis (OA), which is characterised by varying degrees of physical and functional limitation and reduced quality of life, with a major impact on the quality of life of the ageing population in European countries [4]. The cartilage is populated exclusively by chondrocytes; however, its regenerative capacities are limited due to a complicated extracellular matrix (ECM) structure and difficulties associated with repopulating the cells within the tissue after trauma and inflammation [5]. Currently, there is no efficient therapeutic approach for cartilage lesions, and cell-based therapies such as multipotent MSCs from different sources seem promising candidates for cartilage tissue engineering and stimulation of cartilage regeneration [6, 7]. Although the majority of therapeutic techniques using MSCs produce poor outcomes with limited success rates, these cells remain a key focus on studies aimed at differentiating them into a robust chondrogenic lineage and establishing novel protocols for clinical studies.

The main goal of stimulating a qualitative chondrogenic response in cells is to select an appropriate protocol to induce cell cascades responsible for chondrogenesis. One of the major components of all chondrogenic differentiation media is the growth factor transforming growth factor *β* (TGF-*β*), which is crucial for in vivo and in vitro chondrogenesis; however, other factors which also play an important role in this process are not always involved in stimulating MSCs to differentiate. In fact, different tissue MSCs might require novel protocols with different biologically active factors, optimized for a correct tissue source MSCs, which may reveal stronger effects in cell chondrogenic response.

Although MenSCs are known to have a great potential to differentiate into various tissue cells, their chondrogenic differentiation potential has not been extensively investigated so far. In this review, we aim to gather all up-to-date knowledge considering MenSC potential to differentiate into chondrogenic lineage. Currently, BMMSCs have been considered as the most potential candidates for cartilage regeneration techniques; however, these cells deploy a number of disadvantages in their usage, including invasive and painful sample collection, shortage of biological material, and small number of cells in it, whereas, those issues are not relevant to MenSCs.

## 2. MenSC Characteristics

The female reproductive system is a complicated combination of biological components where the uterine endometrium plays an exclusive role. This fast-regenerating tissue has been considered as a source for easy-accessible stem cells decades ago [8]. It is known that the endometrium undergoes over 400 cycles of regeneration and menstruation during a woman's reproductive life cycle, allowing for pregnancy, and can be even continued to regenerate after menopause using estrogen therapy [9]. It was repeatedly confirmed that the endometrium is rich with epithelial progenitor cells as well as MSCs [10–12]. Moreover, endometrium MSCs (EnSCs) have been shown to regenerate into all three different layers—endoderm, mesoderm, and ectoderm—and maintain similar properties to BMMSCs [11, 12]. EnSCs can be isolated directly from the endometrium using hysterectomy or endometrial biopsy; however, these procedures are invasive and require surgical intervention. Another way of collecting EnSCs is their isolation from menstrual blood, which is being naturally discarded from organism each month as waste and requires minimal ethical issues.

**Figure 1:**
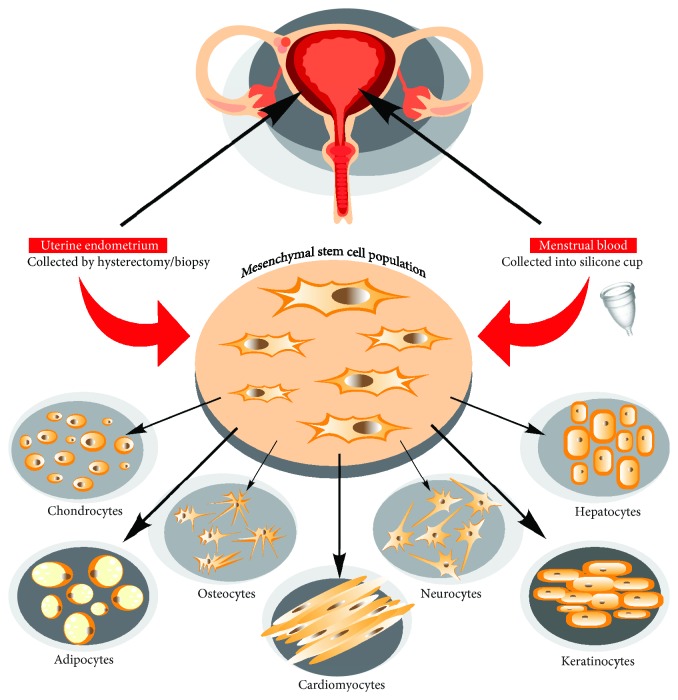
Mesenchymal stem cell isolation from uterine endometrium and menstrual blood and their differentiation potential.

Menstrual blood-derived EnSCs (MenSCs) were firstly observed by Meng and his team in 2007. From that time, this source of collecting MSCs has attracted huge scientific interest, leading to a number of different research avenues and possible applications of MenSCs in clinical practice. It has been shown that MenSCs possess such typical MSC qualities as self-renewal, high proliferative potential, and a multipotent differentiation ability into chondrogenic, adipogenic, and osteogenic lineages in vitro [13], (see Figure 1).

## 3. Differences between BMMSC and MenSC Phenotypes and Differentiation Potentials

BMMSCs are a classical MSC population, which is often employed as a reference control for evaluation of phenotype and functional peculiarities of other sources of MSCs. Although MenSCs share a lot of similar typical properties with BMMSCs, MenSCs seem to have some advantageous characteristics. For instance, recent studies have shown that MenSCs are even able to differentiate into cardiomyocytes with the functions of beating spontaneously after induction resulting in the decreased myocardial infarction area in a rat model [14, 15]. Furthermore, it has been shown that MenSCs are capable to differentiate into neural and epidermal-like cells [16–19] and even functional hepatocytes [20], which suggest superior spectrum of their differentiation potential, as compared to BMMSCs (Figure 1). In addition to the whole range of MSC surface markers, including CD73, CD90, and CD105, MenSCs also express some pluripotency markers, such as OCT-4, SSEA-4 [17, 21], highly upregulated levels of CD49a [22] but lack of STRO1 expression [23, 24], which further distinguishes them from BMMSCs. Furthermore, it has been shown that the proliferation capability of MenSCs is much higher than that of BMMSCs [3, 23, 24]. Colony forming unit (CFU) rate and proangiogenic capacity in vitro have been also established as much higher in MenSCs as compared to BMMSCs [22]. Lower tumorigenicity has been reported for MenSCs, as compared to BMMSCs, which implies safety of MenSC-based therapies [20, 25]. These findings support MenSCs as a unique and promising cell population; however, the beneficial clinical efficacy of those cells in comparison to BMMSCs remains to be investigated.

## 4. Articular Cartilage and Its Regenerative Disability—Stem Cells Might Be an Answer

Articular cartilage, due to its low capacity for self-repair, is highly susceptible to trauma or degenerative low-grade inflammatory diseases such as OA, leading to disability and the loss of quality of life in a considerable part of population worldwide. In 2014, there has been registered more than 237 million (3.3%) of the world's population that are suffering from OA [26]. The prevalence of OA increases with age: 13.9% of adults at age 25 years, while 33.6% of adults at age 65 and older have OA, where more than a half of them are women. This gender difference is important and relevant to the topic of this review. The major factors that increase the risk of OA are age, obesity, gender, joint disease, or abnormalities with its functions, metabolic disorders, and genetic factors [27] but gender is especially important after menopause.

**Figure 2:**
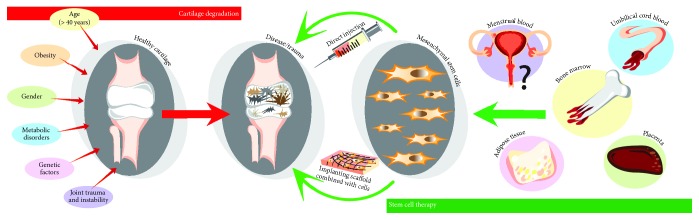
Major OA risk factor strategies for promoting cell-based cartilage repair.

Age is the primary factor for OA, as it usually forms in the 40s onwards. Obesity creates a harmful load on joints and has a negative influence on cartilage, increasing the chance of developing OA and even getting it worse with time [28]. Moreover, according to statistics, OA is most common and severe in women and any kind of surgical operation on a joint can lead to OA [29]. Furthermore, metabolic disorders have also been considered as one of the causes for OA. Altered metabolic pathways and mediators in OA cartilage have been even highlighted as potential therapeutic targets [30]. Equally, alterations in the ion channels that enable Ca^2+^ transport across the plasma membrane seem to be critical for the development of cartilage degeneration in OA [5, 31]. Although all of these factors have been extensively studied, the knowledge has not been translated to therapies—there are still no efficient cell-based therapeutic approaches for cartilage lesions. Cell-based therapies such as multipotent MSCs seem promising candidates for cartilage engineering and regeneration [32]. Tissue engineers have constructed different ways of a possible cartilage treatment with MSCs, including direct injection into cartilage, mixing them with hydrogels, or seeding on scaffolds [33] (see Figure 2). BMMSCs have been identified as the most popular choice for cartilage tissue regeneration techniques due to their plasticity and close location to the cartilage. Furthermore, the placenta, umbilical cord blood, and adipose tissues were also used as MSC sources in cartilage tissue engineering [34, 35].

However, the majority of cartilage engineering or repair techniques using MSCs have failed so far due to a number of complicating factors, such as development of hypertrophy [36]. Hypertrophy is often acquired in MSCs during chondrogenic induction, leading to a possible further differentiation to endochondral bone formation. It is marked by sudden increase in cell volume (more than 10-fold) and structural remodelling of ECM, forming calcification and mineralization of ECM. Cells begin to synthesize collagen type X, produce destructive metalloproteinases. Therefore, hypertrophy affects not only chondrocyte homeostasis but also cartilage structure [37]. Furthermore, there are other MSC application restrictions, as isolating them from a large number of donors, small amount of available cells, and decrease in their proliferation/differentiation rate with age [9].

Menstrual blood is a unique easily accessible source of stem cells, which eliminates the majority of BMMSCs and other tissue MSC restrictions and can be used to treat different diseases, where OA might not be an exception. Although MenSCs were not applied in cartilage regeneration techniques yet, their candidature in these procedures is high. For instance, to the best of our knowledge, there is no data concerning potential formation of fibrocartilage (collagen type I) or hypertrophy (collagen type X, VEGF, MMP-13) during chondrogenesis in MenSCs, which might appear an additional advantage for their application for cartilage repair.

Noteworthily, it is logical to assume that the ability to collect menstrual blood for autologous treatment with MenSCs is progressively reduced in elderly women which could appear a limitation for their therapeutic applications in OA. On the other hand, if these cells could be collected and cryopreserved in advance, there will always be an opportunity to use them later in the donor's lifetime. Moreover, MenSCs are derived from shedding endometrium, suggesting that if the endometrium can maintain its regenerative capabilities even after menopause, this may prolong and sustain stem cell collection, allowing application of autologous biological material in future clinical therapies even for elderly women [25].

## 5. Chondrogenesis and Impact of MSCs

Molecular mechanisms that control chondrogenic differentiation in MSCs have been the major focus of research and important puzzle to solve for exploiting biochemical pathways to induce cartilage regeneration. In vivo chondrogenesis is initiated by several growth factors, such as tumor growth factors-*β* (TGF-*β*s), Activin A, bone morphogenetic proteins- (BMP-) 2, BMP-4, BMP-7, and fibroblast growth factors (FGFs) [38]. TGF-*β* is critical for chondrogenesis as it is considered to be a crucial stimulator for chondrogenic differentiation both in vitro and in vivo [32]. TGF-*β*s (mainly TGF-*β*1 and TGF-*β*3) stimulate chondrogenesis through SMAD3 protein, which further stimulates transcriptional activity of Sox9 leading to activation of cartilage-specific protein genes, as type II and type IX collagen, aggrecan, CD-RAP, and cartilage oligomeric protein (COMP) [39]. FGFs have been shown to promote chondrocyte proliferation in vivo. FGF-2, FGF-9, and FGF-18 are the most studied growth factors in chondrogenesis, where FGF-2 upregulates Sox9 and early activation of chondrogenesis and FGF-9/18 maintain chondrocyte phenotype, delaying hypertrophy [36, 40]. Furthermore, FGFs often act in concert with other growth factors such as insulin-like growth factors (IGFs) that are required for a proper chondrogenesis formation, as well as cell proliferation and motility. IGF-1 was found to be equally potent to TGF-*β*1 in chondroinductive actions of BMMSCs (Longorbardi et al., 2006). Moreover, it enhances cartilage matrix formation, regulates apoptosis, and blocks interleukin-1-induced turnover of proteoglycans in chondrocytes, which makes this factor an important element in chondrogenesis (Chun du oh, 2003). Wingless proteins (Wnts) are important in a variety of cellular activities during chondrogenic differentiation, including proliferation and gene expression, as they induce production of FGFs [41–43]. Sonic hedgehog (SHH) induces MSCs to synthesize BMPs, directing MSC differentiation into chondrogenic lineage [44]. Furthermore, several factors maintain the chondrocyte phenotype in the cartilage, such as parathyroid-related peptide (PTHRP) and Indian hedgehog (IHH) [44].

**Figure 3:**
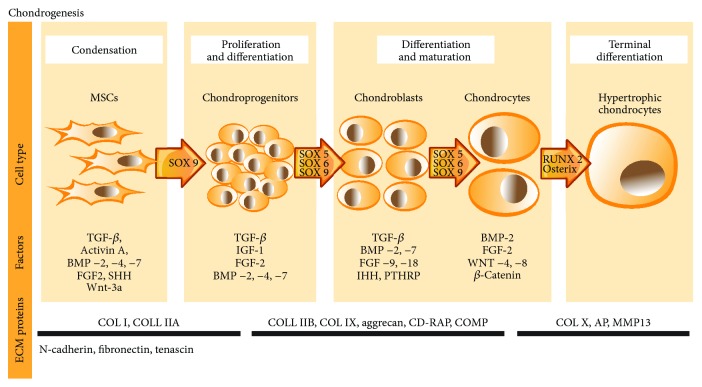
Stages of chondrogenesis in vivo.

All of these growth factors play a key role in tissue repair and regeneration, and most importantly—these are crucial factors in all chondrogenesis stages [45, 46] (see Figure 3). Transcription factors also play an essential role in chondrogenesis as they regulate not only the expression of ECM proteins but also the expression of growth factors according to the differentiation stage. Sox9 is one of the earliest markers expressed in the MSCs and is the key transcription factor in chondrocyte maturation [47]. Sox5 and Sox6 maintain chondrocyte phenotype at later stages and directly regulate expression of ECM molecules, such as collagen (IIB, IX, X) and proteoglycans (aggrecans) [48]. RunX2 and osterix negatively affect chondrogenesis, as they induce mineralization of the cartilage matrix [49], by promoting matrix metalloproteinase 13 (MMP13) synthesis [50]. Generally, MMP synthesis in cells is stimulated by proinflammatory cytokines, allowing them to negatively regulate cell processes. In the cartilage, MMPs (mainly MMP-9, MMP-10, MMP-13, and MMP-14) lead chondrocytes to hypertrophy and remodel ECM, forming cartilage degradation [44].

In the meantime, classical chondrogenic differentiation medium consists of a combination of growth factors (predominantly TGF-*β*s), ITS, high-glucose, dexamethasone, ascorbic acid-phosphate, sodium pyruvate and proline, and in major cases—lacks serum. These factors along with natural stem cells secreting biologically active compounds stimulate their differentiation towards chondrogenic lineage. For this reason, before applying stem cells in tissue regeneration techniques, it is useful and important to evaluate their secretome profile.

MSCs are beneficial for OA repair techniques due to their anti-inflammatory and chondroprotective properties. They are known to secrete a broad range of various paracrine factors and bioactive molecules that can modulate metabolism of extracellular matrix in OA cartilage [7]. Cytokines are major factors that regulate cell differentiation capabilities. BMMSC secretome was characterised in many studies. It was found that BMMSCs secrete a wide range of different cytokines/growth factors, including interleukins: IL-6, IL-7, IL-8, IL-11, IL-12, IL-14, IL-15, leukemia inhibitory factor (LIF), granulocyte colony-stimulating factor (G-CSF), granulocyte macrophage colony-stimulating factor (GM-CSF), macrophage colony-stimulating factor (M-SCF), Flt-3 ligand (FL), and stem cell factor (SCF) [51].

## 6. Growth Factors Secreted by MenSCs and Their Potential Impact on Chondrogenic Differentiation

As for MenSCs, their secretome is less studied; however, several studies already published their results according to MenSC cytokine and growth factor secretion, cultivating them in monolayer (see Table 1).

It has been observed that among basal proliferative, angiogenetic, and chemo-attractive proteins, such as VEGF, PDGF, HGF, and ANG-2, MenSCs secrete biologically active molecules IGF-1 and FGF-2, which are involved in different stages of chondrogenesis (Figure 3) [25, 53], as described earlier. Furthermore, MenSCs express Activin A, which is a member of the TGF-*β* superfamily. Several studies suggest that Activin A plays a pivotal role in the early stages of MSC chondrogenesis [55, 56]. Activin A induces the expression of Oct4, Nanog, Nodal, Wnt3, and FGF8 and is necessary for the maintenance of self-renewal and pluripotency of MSC [55]. Enhanced production of Activin A was demonstrated in OA cartilage, associated with the suppression of aggrecanase-mediated cleavage of aggrecan in human articular cartilage [57], suggesting a chondroprotective role of Activin A during destructive OA process. Chimeric ligands of Activin A and BMP-2 have been used to induce chondrogenic differentiation in adipose tissue-derived MSCs (ASCs) resulting in Peran et al. [56]. They demonstrated increased expression of collagen type 2, Sox9, and aggrecan in ASCs (toluidine blue and Masson's trichrome staining), which was also confirmed by RT-PCR in response to Activin A/BMP-2 chimeras [56]. Besides, Activin A is involved in regulation of women menstrual cycle [58] suggesting that it may appear pivotal for modulation of MenSC differentiation potential. Our preliminary data also confirmed chondrogenic differentiation capacity of MenSCs and its modulation by Activin A (unpublished data).

Conversely, MenSCs secrete immunomodulating factors as IL-6, IL-8, IL-10, IFN-*γ*, GRO, OPG, HGF, and MCP-1, which take place in an inflammatory process. These factors were studied according to immunosuppressive properties of MenSCs and BMMSCs, which were analyzed in collagen-induced arthritis model in mice and their secreted factors, activated with/without IFN-c and IL-1b. The study concluded that MenSCs are less responsive to cytokine activation and express less immunosuppressive molecules compared to BMMSCs [22], which is not an advantageous fact if considering their applicability in cartilage regeneration. Moreover, MenSCs are shown to express matrix metalloproteases (MMP-3, MMP-10) [3]. The secretion of these factors is considered to negatively affect chondrogenesis in these cells, as they promote chondrocyte hypertrophy, as described earlier.

Nevertheless, these are only few studies made in the field of MenSC secretome. This niche requires more studies to truly understand the nature of these cells and their secreting factors, which can possibly approve or disprove already published results.

## 7. MenSC Chondrogenic Differentiation Capability for Tissue Engineering Approaches

MenSCs are known to have a great potential to differentiate into various tissue cells; however, their chondrogenic differentiation potential has not been extensively investigated so far. The primary study which analyzed ESC chondrogenic differentiation potential in time was made in 2007 by Wolff and colleagues [59]. They reported that ESC pellets cultured in chondrogenic media secreted proteoglycan as the extracellular matrix was stained with Alcian blue, while control pellets were cultivated in chondrogenic media without growth factors and in DMEM did not. They concluded that endometrial stem cells are capable to differentiate into chondrogenic lineage and that there is a coherence between the staining intensity and differentiation time; for instance, as longer pellets were differentiated, the more proteoglycan were accumulated. In Table 2, we have summarized data from all published studies in the field of MenSC chondrogenic differentiation, including the exact methodologies used by the authors including growth factors and differentiation duration.

According to these studies, MenSCs revealed different results in chondrogenic response. Several published studies suggest that MenSCs could be a suitable candidate for cartilage tissue engineering and may have direct effects on cartilage tissue repair, as determined by sulfated glycosaminoglycans and express collagen type II [20, 21]. Other authors suggest that MenSCs have low chondrogenic differentiation potential and are not a suitable stem cell population for cartilage regeneration. For instance, in 2015, there was a study published where authors compared gene expression between MenSCs and umbilical cord MSCs (UCMSCs) from the same donor and between MenSCs and BMMSCs from the same donor. They screened 768 genes in MenSCs, UCMSCs, and BMMSCs. Furthermore, they report that important osteogenic and chondrogenic genes POSTN and OSTM1 were largely downregulated in MenSCs compared with UCMSCs and BMMSCs, which also confirmed the inferior osteogenic and chondrogenic differentiation potentials of MenSCs [18]. However, these authors did not induce their cells to differentiate, which is uncertain due to changes in chondrogenic genes during differentiation process. POSTN gene codes periostin, which was shown to promote osteogenic differentiation [63], inducing ECM mineralization but not chondrogenic differentiation [64]. Furthermore, the expression of these genes in cells is upregulated during differentiation process, so it is unclear what the true expression of these genes is during chondrogenic induction.

On the other hand, the majority of authors claim that differentiated MenSCs (ESCs) showed strong immunoreactivity to a monoclonal antibody against Collagen type 2 and accumulation of proteoglycan that were revealed by Alcian blue staining [24, 59, 60, 62], which are believed to be considerable confirmation of chondrogenesis. Moreover, the comparison of differentiated MenSCs and BMMSCs showed a similar pattern of proteoglycan accumulation [24]. However, the expression of Collagen 2A1 mRNA was particularly observable in differentiated BMMSCs, although not in MenSCs [24], which can be related to inappropriate growth factor induction, which the authors used—TGF-*β*3, BMP-6. Nevertheless, during MenSC differentiation, they detected a significant increase in the expression level of Collagen 9A1 and the transcription factor SOX9, suggesting that these cells positively respond to chondrogenic induction. Moreover, considering different growth factor influence on chondrogenic differentiation, it is important to note that there are studies suggesting that TGF-*β*3 does not always induce chondrogenesis in such cells as ADSC and BMMSCs, where BMP-2 was shown to act as a major chondrogenic differentiation inducer in ADSC [65, 66], and the combination of TGF-*β*1, GDF-5, and BMP-2 stimulated robust chondrogenic response in BMMSCs [67]. These observations may lead to the development of new strategies for novel chondrogenic differentiation protocols for MenSCs, which will include additional factors that these cells may require. For instance, Activin A is known to be crucial in the early stages of chondrogenesis, as described earlier; however, classical chondrogenic differentiation medium does not contain it. Additional growth factors might be useful in MenSC differentiation capability.

## 8. Conclusions

Menstrual blood is a unique body fluid that contains multipotent cells with typical characteristics of MSCs, while with a greater proliferative and differentiation capability than classical bone marrow-derived MSCs (BMMSCs). These advantages, as well as the ease of access of MenSCs due to possibility of repeated noninvasive menstrual blood sample collection even from the same donor, make MenSCs a promising cellular source for regenerative medicine applications.

Although these cells have many more benefits comparing to other tissue MSCs, some of the research niches still need further investigation to fully identify the applicability of MenSCs for basic research and clinical applications. One of these niches is their chondrogenic differentiation. As articular cartilage has difficulties in self-regeneration and is susceptible to OA, especially in women, MenSCs could serve as a perfect stem cell therapy tool for cartilage regeneration. However, the chondrogenic differentiation potential of MenSCs remains controversial. One concept and claim is that these cells have a strong potential to differentiate, as they efficiently produce proteoglycans and collagen type II [20, 22–24, 62] and may have direct effects on cartilage tissue repair. Another concept is that MenSCs have a weak chondrogenic response [18]. Induction of relevant differentiation cascades in those cells by stimulating them with adjusted set of appropriate growth factors may result in efficient chondrogenic differentiation. However, those issues remain unresolved and require thorough investigation. Taken together, the application of MenSCs for chondrogenic differentiation can provide important information about cartilage function and repair potential and may possess significant regenerative value both as a tool for cartilage tissue engineering and for intra-articular cellular therapy based on stimulating paracrine effects. We conclude that these cells might become a realistic and attractive alternative for cartilage regeneration.

## Figures and Tables

**Table 1:** The analysis of MenSC secretome in published studies^∗^.

Analyzed cytokines/growth factors	MenSCs positive for	Conclusion	Reference
MMP-3, MMP-10, GMCSF, PDGF-BB, ANG-2, VEGF, HGF, EGF	MMP-3, MMP-10, GM-CSF, PDGF-BB, ANG-2, VEGF, HGF, EGF	MenSCs share some properties of mesenchymal stem cells based on phenotype but functionally produce factors that are unique.	[3]
VEGF, BDNF, GDNF, NT-3	VEGF, BDNF, NT-3	Oxygen glucose deprivation (OGD) conditions showed upregulation of VEGF, BDNF, and NT-3 in MenSCs, comparing to normal condition cultivation.	[8]
IL-10, IFN-*γ*, MCP-1, IDO1, COX-2, FOXP3	IDO1, COX-2, FOXP, IFN-*γ*, IL-10, MCP-1	MenSCs from patients with endometriosis express higher amounts of IDO1, IFN-*γ*, MCP-1, and IL-10.	[52]
Activin A, IL-6, Cox2, IDO, PDL-1	IL-6, Cox2, Activin A, IDO, PDL-1	MenSCs are less responsive to cytokine activation and express less immunosuppressive molecules compared to BMMSCs.	[22]
VEGF, HGF, IGF-1	VEGF, HGF, IGF-1	MenSCs make a significant stem cell population, producing cytokines, crucial for tissue repair and regeneration.	[53]
VEGF, FGF, KGF, HGF	VEGF, FGF-2, KGF, HGF	MenSCs secrete higher concentration of HGF than from dental pulp—MSCs at the sixth and tenth passage and had the lowest concentration in FGF (from P2 to P10).	[25]
MCP-1, IL-6, HGF, GRO, IL-8, OPG	MCP-1, IL-6, HGF, GRO, IL-8, OPG	MenSCs have a potential for reducing liver fibrosis in mice.	[54]

^∗^Abbreviations: BDNF: brain-derived neurotrophic factor; Cox: cyclooxygenase; EGF: epidermal growth factor; FGF: fibroblast growth factor; FOX: forkhead transcription factor; GDNF: glial cell line-derived neurotrophic factor; GMCSF: granulocyte macrophage colony-stimulating factor; GRO: growth-related oncogene; HGF: hepatocyte growth factor; IDO: indoleamine 2,3 dioxygenase; IFN: interferon; IGF: insulin-like growth factor; IL: interleukin; KGF: keratinocyte growth factor; MCP: monocyte chemoattractant protein; MMP: metalloprotease; NT: neurotrophin; ANG: angiogenic factor; OPG: osteoprotegerin; PDGF: platelet-derived growth factor; PDL: programmed cell death-ligand; VEGF: vascular endothelial growth factor.

**Table 2:** Evidence of MenSC chondrogenic differentiation.

Method	Visualization with	Growth factors/other components used	Duration	Results	Reference
2D	Alcian blue	TGF-*β*3	14–20 days	Alcian blue positive	[60]
	Alcian blue, RT-PCR for collagen type II and Sox9	TGF-*β*3, BMP-6, fibronectin-coated	21 days	Alcian blue positive, collagen type 9 and Sox9 positive, collagen type II negative	[24]
	IHC collagen type II antibody	TGF-*β*3, BMP-6	21 days	Collagen type II positive	[52]
	Alcian blue	TGF-*β*3	14 days	Alcian blue positive	[20]
	Alcian blue	TGF-*β*3	21 days	Alcian blue positive	[53]

3D	Alcian blue, IHC with collagen type II antibody	TGF-*β*3, IGF-1, nanofibrous scaffolds	4 weeks	Alcian blue and collagen type II positive	[11]
	Alcian blue, IHC with collagen type II and type I antibodies	TGF-*β*3, nanofibrous scaffolds	3 weeks	Alcian blue and collagen type II positive, collagen type I negative	[23]
	Alcian blue	TGF-*β*, BMP-6	21 days	Alcian blue positive	[61]
	IHC with collagen type II antibodies	TGF-*β*3, IGF-1	4 weeks	Collagen type II positive	[62]
	Safranin O, collagen type II RNA gene analysis	TGF-*β*3	21 days	Safranin O and collagen type II gene positive	[22]
